# Microtubule plus-ends reveal essential links between intracellular polarization and localized modulation of endocytosis during division-plane establishment in plant cells

**DOI:** 10.1186/1741-7007-3-11

**Published:** 2005-04-14

**Authors:** Pankaj Dhonukshe, Jaideep Mathur, Martin Hülskamp, Theodorus WJ Gadella

**Affiliations:** 1 Section of Molecular Cytology, Swammerdam Institute for Life Sciences, University of Amsterdam, Kruislaan 316, 1098 SM Amsterdam, The Netherlands; 2 Department of Plant Agriculture, Molecular Cell Biology Laboratory, University of Guelph, Guelph, ON, N1G 2W1, Canada; 3 Botanical Institute III, University of Köln, Gyrhofstrasse 15, Köln, 50931, Germany; 4 Centre for Molecular Biology of Plants, University of Tübingen, Auf der Morgenstelle 3, 72076 Tübingen, Germany

## Abstract

**Background:**

A key event in plant morphogenesis is the establishment of a division plane. A plant-specific microtubular preprophase band (PPB) accurately predicts the line of cell division, whereas the phragmoplast, another plant-specific array, executes cell division by maintaining this predicted line. Although establishment of these specific arrays apparently involves intracellular repolarization events that focus cellular resources to a division site, it still remains unclear how microtubules position the cell division planes. Here we study GFP-AtEB1 decorated microtubule plus-ends to dissect events at the division plane.

**Results:**

Early mitotic events included guided growth of endoplasmic microtubules (EMTs) towards the PPB site and their coincident localization with endocytic vesicles. Consequently, an endosomal belt lay in close proximity to the microtubular PPB at its maturation and was maintained during spindle formation. During cytokinesis, EMTs radiated from the former spindle poles in a geometrical conformation correlating with cell-plate navigation and tilt-correction. Naphthylphtalamic acid (NPA), an inhibitor of polar auxin efflux, caused abnormal PPBs and shifted division planes.

**Conclusion:**

Our observations reveal a spatio-temporal link between microtubules and intracellular polarization essential for localized endocytosis and precise establishment of the division plane in plants. Additionally, they implicate the growth regulator, auxin, in this important cellular event.

## Background

In cell wall-encased, immobile plant cells, tight regulation of the cell division plane plays a crucial role in tissue and organ morphogenesis [1,2]. At the onset of mitosis a plant-specific cortical microtubule array, the PPB [3], emerges. Although the PPB disassembles as the cell enters mitosis, it precisely predicts where the new cell plate attaches to the parental cell walls at the end of cytokinesis [1,2]. Cell division planes are drastically affected by the absence of PPB [4], which may be achieved through experimental obliteration [5] or through genetic defects [6]. Since the discovery of the PPB four decades ago, the mechanism governing its creation and effect on delineating the future cell division plane has remained a mystery.

Though initially considered to be a cortical process, PPB formation involves two coincident cytoplasmic events too, namely the migration of the nucleus towards the cell center [7], and recruitment of numerous EMTs within motile cytoplasmic strands [8,9]. In centrifuged protonemata (consisting of a single elongated cell), nuclear displacement prior to pre-prophase induces the formation of a PPB around the new nuclear position, suggesting a role for the nucleus in PPB placement [10]. However, in plant cells under normal gravity conditions, the nucleus is positioned by microtubules, since its random migration is prevented by microtubular depolymerization [11]. These observations point towards a functional connection between the nucleus, microtubules and cell cortex for marking the PPB. In budding yeast, EMTs play a role in bringing the nucleus to the division plane by a cortical 'search and capture' mechanism [12-14]; and in fission yeast, EMTs position the nucleus by pushing against the cortex [15]. Under *in vitro*, simulated conditions, the microtubule seeds also probe the metallic boundaries of artificial chambers with cellular dimensions to bring nuclei to the center [16]. Together, these studies on diverse systems corroborate the role of microtubules in probing the cellular geometry to settle the nucleus at the center of the cell. In the absence of information on the polarity and dynamics of plant EMTs, their precise role in association with the nucleus and the PPB is unclear.

In plants, in addition to the PPB, the other two mitotic microtubular arrays, the spindle and the phragmoplast microtubules, are also of endoplasmic nature. During the spindle stage, the perpendicularity of the spindle to the cell division plane is often lost because of its rotation. In mammalian and yeast cells the spindle is kept at the center of the cell or at the bud site by the centrosome-originating astral microtubules [17,18]. In addition, any error in spindle orientation in these cell types is corrected by the outgoing astral microtubules, which probe the cortex to ensure that the segregated chromosomes are sufficiently distant and are not cleaved by the constricting actomyosin ring [19,20]. In acentrosomal plant cells, after PPB breakdown and disappearance of the nuclear surface-bound EMT, the factors maintaining the spindle at the cell centre still remain unresolved. Paradoxically, in plant cells possessing disoriented spindles, the cell plates are still able to anchor properly to the division sites marked perpendicularly to the cell axis. A mechanism obviously exists in plant cells to correct spindle disorientation and reinforce the line of cell division, but it remains obscure.

Here, we investigated the role of EMTs during PPB formation and their subsequent behavior following karyokinesis. Because of their uniform cell size, continuous cell division activity and absence of background fluorescence, Tobacco BY-2 cell suspensions remain a system of choice for plant cell cycle studies [21]. Therefore, we used tobacco BY-2 cells stably transformed with different microtubular markers, and performed a live cell time-lapse analysis to elucidate the role of EMTs in reinforcing the lines of the cell division planes in plants.

## Results

### GFP-AtEB1 labeled plant microtubule plus ends exhibit guided growth to create bundles

**Figure 1:**
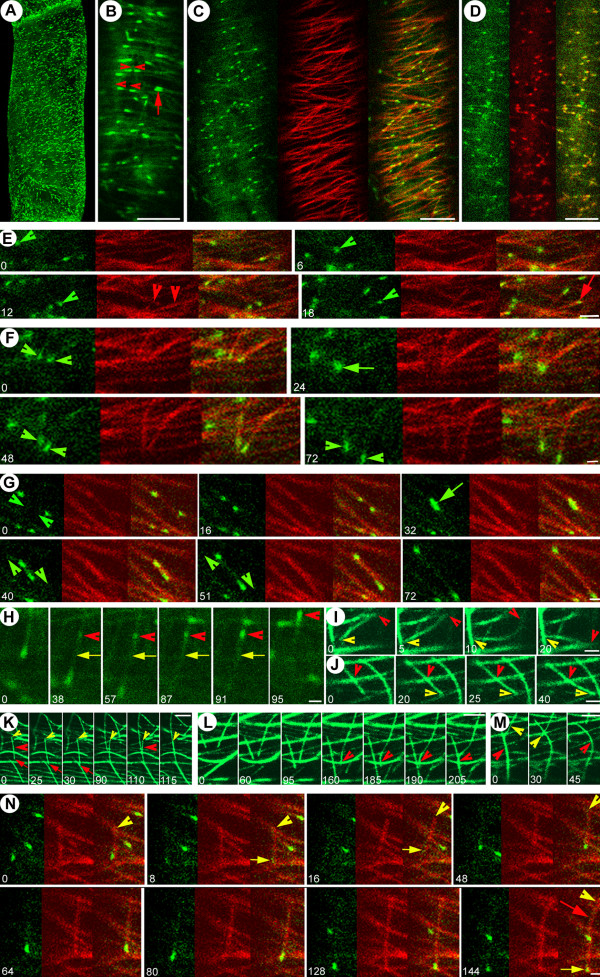
**Mechanisms for microtubule guidance and bundling**. Green: GFP-AtEB1 (in A-H and N) and GFP-MAP4 (in I-M). Red: YFP-MAP4 (in C, E-G and N) and YFP-CLIP170 (in D). (A) 3-D maximum projection of interphase BY-2 cell, highlighting punctuate GFP-AtEB1 labeling in the cortex. (B) In interphase cells, GFP-AtEB1 comets at the cortex move in the same or opposite direction on the same track (arrowheads), and sometimes together (arrow) (See additional file 1: Movie 1). (C) In co-transformed cells, GFP-EB1 labels the growing ends of microtubules labeled by YFP-MAP4 (See additional file 2: Movie 2). (D) Co-localization of GFP-AtEB1 and YFP-CLIP170 on growing microtubule plus ends (See additional file 3: Movie 3). (E) GFP-AtEB1 labeled growing microtubule changing from one microtubule track (labeled with YFP-MAP4) to another. [58] Growth of two separate unbundled microtubules (arrowheads) transiently meeting (arrow) and afterwards separating without inducing catastrophe. (G) Microtubules growing in opposite directions on the same track with similar speed (arrowheads) meet (arrow) and continue growing in opposite directions without inducing catastrophe. (H) Microtubule nucleation and growth (arrowheads) on an already existing microtubule (arrow). (I) Situation where attachment of a microtubule plus end (yellow arrowhead) to an existing microtubule induces a translocation of the minus end (red arrowhead) from one microtubule to another. (J) Microtubule plus end (yellow arrowhead) growing towards an existing microtubule, followed by guided growth in a new direction, causes bending at the point of previous attachment (red arrowhead). (K) Treadmilling microtubule (red arrowhead) with its plus end (red arrow) and minus end (yellow arrowhead) moving in the same direction (yellow arrowheads and red arrows) initiates bundling by bridging two other separate preexisting microtubules. (L) Long microtubule growing (arrowhead) and interacting with an existing microtubule induces reorientation of growth and bending, resulting in bundling with a shorter growing microtubule. (M) Depolymerization of one microtubule partially associated with a bundle (yellow arrowhead) causes bending of the remaining structure (red arrowhead). (N) Microtubule minus end detachment and subsequent movement (arrowheads) induces loss of GFP-AtEB1 from its plus end (yellow arrows); it recovers GFP-AtEB1 labeling and plus end growth once its minus end acquires new support on another polymer (arrowhead). Note that the same microtubule bends (red arrow) when its minus end is fixed and the plus end (yellow arrow) hits another microtubule. Time is indicated in seconds and bars represent 5 μm in A-D, 3 μm in K-M, 2 μm in E, I, J and 1 μm in F-H, N.

Microtubule plus-end labeling has been achieved using a GFP-AtEB1 fusion protein and permits observations on microtubule growth directionality and dynamics [22]. In interphase cells, GFP-AtEB1 highlighted the bidirectional movement of comet-like structures, suggesting plus end growth of cortical microtubules of opposite growth polarities (Figure 1A, B; see also additional file 1: Movie 1). In co-transformed cells, GFP-AtEB1 labeled the growing ends of YFP-MAP4 labeled microtubules (Figure 1C; see also additional file 2: Movie 2) and co-localized with YFP-CLIP170 (Figure 1D; see also additional file 3: Movie 3) on the growing microtubular plus ends, reconfirming the plus end-specific localization of GFP-AtEB1 in BY-2 cells.

General observations on microtubule guidance and bundling formed the basis of our subsequent experiments. Tracking the GFP-AtEB1 comets, it was found that >80 % of freshly polymerizing microtubules exhibited guided growth on tracks established by existing microtubules, thereby creating bundles of microtubules (Figure 1C; see also additional file 2: Movie 2). Preexisting microtubule-guided oriented bundling often involved i) two or more microtubules with apparently similar polarity moving one after the other, ii) microtubules with presumably opposite polarities moving in opposite directions to each other or iii) independent microtubules moving together as a pair with similar velocities (4.15 ± 0.41 μm/min, n = 29) (Figure 1B; see also additional file 1: Movie 1). Although mammalian EB1 often induces microtubule bundling upon overexpression, AtEB1 did not cause the observed bundling in the plant cells since it lacks the microtubule bundling domain present in the mammalian ortholog [23]. Instead, intermicrotubular bridges [24] might be responsible for the observed bundle formation. In addition, when two microtubules grew together side-by-side on the same track, it was frequently observed that one of them shrank while the other continued growing (see additional file 1: Movie 1). Further information on microtubular guidance and bundling mechanisms came from analysis of cells coexpressing GFP-AtEB1 and YFP-MAP4. A growing microtubule (green arrow, Figure 1E) could detach from an existing track and move on to another track where its growth became guided in another direction (Figure 1E). Interphase microtubules in mammalian cells show a similar guidance mechanism [25]. We also observed individual microtubule plus ends approaching each other from a similar (Figure 1F) or opposite (Figure 1G) direction and meeting without inducing catastrophe. Strikingly, microtubular nucleation was sometimes initiated on an existing microtubule (Figure 1H), an observation consistent with the plant-specific localization of gamma tubulin along the microtubule length [26] and with a study reporting microtubule nucleation from stable tubulin oligomers [27]. Microtubules also changed trajectories by reorienting their minus or plus ends when one of the ends was supported on the other polymer (Figure 1I, J). It was also observed that motile polymers exhibiting specialized treadmilling [28] initiated bundling by bridging the two preexisting and separate polymers (Figure 1K). In cases of guided growth-induced bundling, a shorter microtubule could adopt the growth direction of a preexisting longer microtubule and vice versa (Figure 1L). When one of the microtubules in a bundle retracted, it frequently caused the other to bend, implying the exertion of a pulling force (Figure 1M). We also observed that upon release of a minus end from a nucleation site, the opposite plus end depolymerized (with concomitant loss of GFP-AtEB1), whereas upon acquisition of a new support by its minus end the microtubule retained growth (and regained GFP-AtEB1) (Figure 1N). These observations indicate a provision for new nucleation on existing polymers, while suggesting that in certain cases the plus end somehow 'senses' the physical state of the opposite minus end. Together, these findings implicate intermicrotubular affinities and the capacity of the polymers to nucleate new or detached microtubules as a general mode of microtubule survival, reorientation and bundle creation.

### Emergence, polarity and dynamics of EMTs at the onset of cell division

**Figure 2:**
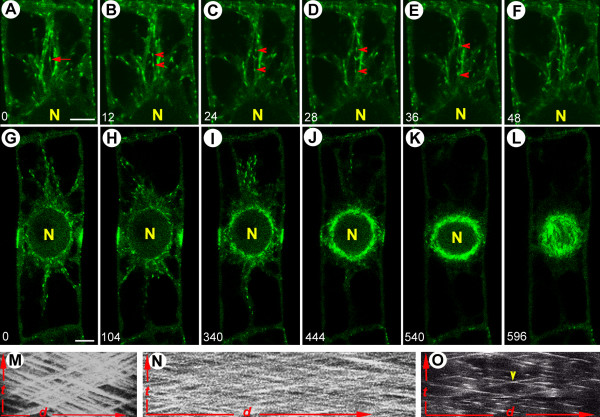
**Polarity and growth speed of EMTs bridging nucleus and cortex**. Green: GFP-AtEB1 (in A-L). (A-F) EMTs exhibit bidirectional growth and microtubule bundling. Note that that the microtubule originating from the nuclear surface (outgoing) and the one coming from the cortex (incoming) cross each other (arrow) and, as in the cortical array, grow with similar speeds without interfering each other (arrowheads) (see additional file 4: Movie 4). (G-L) EMT plus ends radiating mainly in an outward direction from the NE during PPB maturation (see additional file 5: Movie 5). Kymograph projection of microtubule plus ends in the interphase cortex (M), PPB cortex (N) and preprophase cytoplasm (O) showing sustained polymerization. The horizontal axis, *d*, represents distance (18 μm in M, 13 μm in N and 20 μm in O), and the vertical axis, *t*, represents time (290 s in M, 140 s in N and 390 s in O). Note that for each of the 3 cases (M-O), the microtubules follow the tracks, exhibit bi-directionality and grow with the same speeds. By comparing the slopes between images M-O, it becomes evident that the microtubule growth speed increases from interphase to the PPB stage, as previously reported [9]. Note that the arrowhead in M shows the crossing of two EMTs growing on the same path at the same time but in opposite directions. Nucleus is marked by 'N', time is indicated in seconds and bars represent 8 μm.

Equipped with information on the general polar behavior of microtubules in interphase cells we approached the questions of appearance, polarity and dynamics of the EMTs, specifically at the onset of cell division. During preprophase, more dynamic EMTs emerged, bridging the nucleus to the cortex and exhibiting considerable bidirectionality (Figure 2A–F; see also additional file 4: Movie 4) with outgoing (from the nucleus towards the cortex) and incoming (from the cortex towards the nucleus) EMTs. More outgoing (80%) than incoming microtubules were observed though their growth rates were similar (5.86 ± 0.82 μm/min, n = 15 – outgoing; and 5.56 ± 0.47, n = 15 – incoming). Like cortical microtubules, EMTs also exhibited bundling and guidance characteristics indicating the existence of intermicrotubular affinities in the cytosol even in the absence of a cortical support. In contrast to yeast preprophase cells, where unidirectional microtubules (growing from the center towards the cell periphery) position the nucleus by pushing or pulling forces [12,15], our observations together with others [11] on the requirement of EMTs for nuclear displacement and their bidirectionality suggest that plant cells can utilize both outgoing and incoming EMTs for positioning the premitotic nucleus. Conversely, with bundling and track follow-up, the incoming microtubules might guide the outgoing ones to achieve selective cortical targeting. This may be an efficient mechanism for their navigation of intracellular space, since when many microtubules grow simultaneously in a bundle, the chance that microtubules will reach the cortical target(s) without becoming depolymerized in the process is expected to be substantially higher. Interestingly, EMTs maintained continuous contact with the cortical areas occupied by the developing PPB. At PPB maturation, the EMTs between the PPB and nuclear envelope (NE) remained bidirectional while those connecting to more distal cortical areas became unidirectional, displaying a radiating comet-like spectacular firework (Figure 2G–L; see also additional file 5: Movie 5). At this stage, kymographs generated by tracking the GFP-AtEB1 comets clearly illustrate accelerated growth for both outgoing (8.33 ± 0.83 μm/min, n = 30) and incoming (8.02 ± 1.15 μm/min, n = 12) EMTs. Microtubule growth was maintained at ca. 6.78 ± 0.89 μm/min (n = 50) at the PPB [9] (Figure 2M–O). Consequently, the microtubule density on the NE gradually increased (see Figure 2G–K), confirming earlier observations on microtubular growth and stabilization on the NE in plant [9] and mammalian [29] cells. Our observations suggest that at the onset of mitosis, the outwardly-radiating EMTs position the nucleus in the center of the cell by pushing/pulling forces, while the bidirectional EMTs connecting the NE to the PPB position the nucleus at the centre of the PPB. Moreover, during PPB maturation, the change from bidirectional growth (from the distal cortex towards the NE and from the NE towards the distal cortex) to unidirectional growth (from the NE towards the distal cortex) of EMTs severely reduces their chance of survival and thereby causes their detachment and collapse.

### Role of EMTs in premitotic cytoplasmic organization

**Figure 3:**
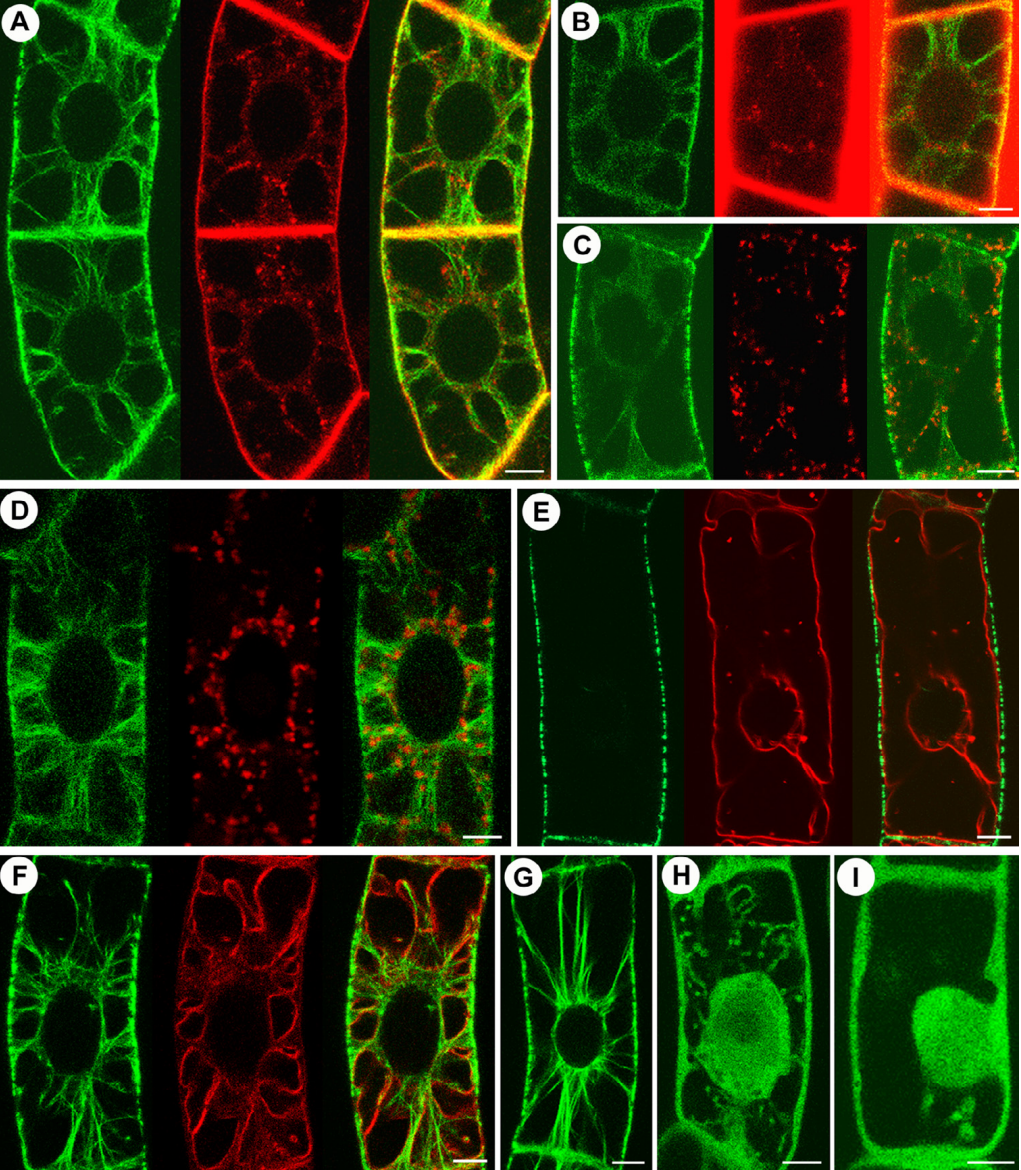
**EMTs configure the premitotic cytoplasm**. Green: GFP-MAP4 (in A-I). Red: FM4-64 (in A, E and F), Alexa 633 (in B), ST-YFP (in C), Mitotracker (in D) FM4-64 labeled endosomes (A), Alexa 633 labeled pinocytic vesicles (B), ST-YFP labeled GA (C) and Mitotracker labeled mitochondria (D) all remain in the vicinity of GFP-MAP4 marked EMT tracks. (E) At interphase, GFP-MAP4 labeled microtubules remain in the cortex and FM4-64 labeled vacuoles occupy most of the endoplasmic space. [F] At preprophase, GFP-MAP4 labeled EMTs intersect the vacuoles labeled by FM4-64. Premitotic cells treated with latrunculin B (G), oryzalin (H) or both (I) show cytoplasmic disorganization in the presence of oryzalin (H-I). Bars represent 8 μm.

The implications of the EMT configuration for the organization of the premitotic cytoplasm were now investigated using GFP-MAP4 transformed BY-2 cells [9] together with various organelle markers. During G2-M transition, FM4-64-labeled endosomes (Figure 3A), Alexa 633-labeled pinocytic vesicles (Figure 3B), ST-YFP-labeled Golgi bodies (GA) (Figure 3C) and Mitotracker-labeled mitochondria (Figure 3D) all localized along the EMTs. In contrast to interphase, when the microtubules remain at the cortex and large vacuoles occupy the cell space (Figure 3E), the vacuoles appeared fragmented by intersecting EMTs during preprophase (Figure 3F). Previous studies have shown that the motility of cytoplasmic organelles in plants is mainly actin-based [30] and that the actin cytoskeleton co-exists with the mitotic microtubular arrays [31]. To further investigate the respective roles of microtubules and actin filaments in premitotic cytoplasmic organization, we treated the cells with latrunculin B (an actin polymerization inhibitor) and oryzalin (a microtubule depolymerizing herbicide). In latrunculin B-treated cells, the EMTs appeared stabilized and more intense with a normal cytoplasmic configuration (Figure 3G), whereas oryzalin destroyed the EMTs and caused cytoplasmic disorganization and nuclear displacement (Figure 3H). After combined oryzalin and latrunculin B treatment the nucleus completely lost its central position and the cytoplasm (stained with unbound GFP-MAP4) became completely disorganized (Figure 3I). After the oryzalin was washed out, the EMTs gradually reappeared and the cytoplasm regained its normal configuration with the nucleus replaced at the cell centre (data not shown). Together, these results suggest that EMTs have a major role in organizing the premitotic cytoplasm, but they do not discount the role of actin in mediating organelle motility.

### Guided growth of EMTs towards the PPB site and their coincident localization with endocytic vesicles

**Figure 4:**
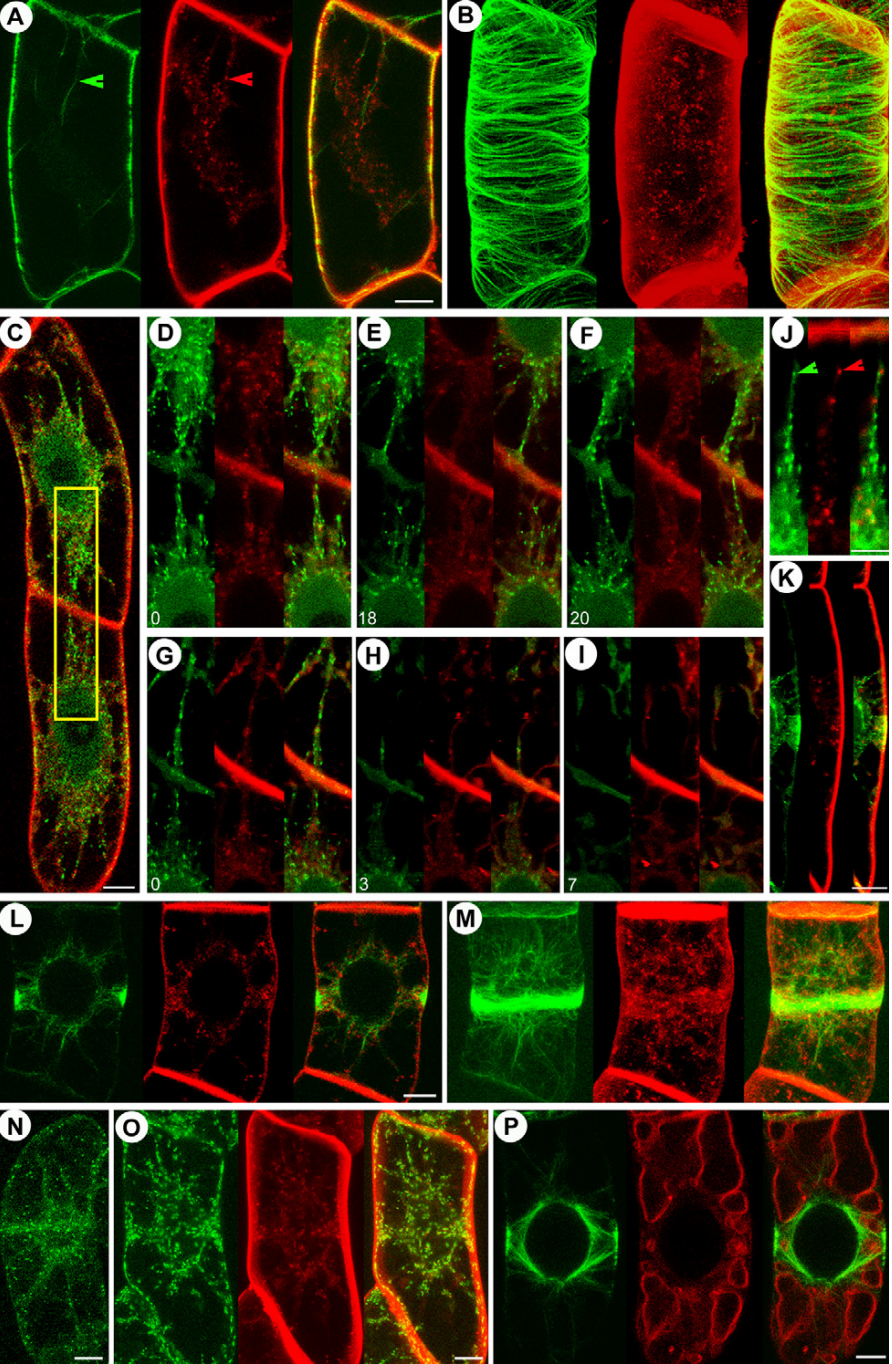
**Endosomal belt co-localizes with microtubular PPB during preprophase**. Green: GFP-MAP4 (in A, B, L, M and P), GFP-AtEB1 (in C-K), GFP-Ara7 (in N) and ST-YFP (in O). Red: FM4-64 (in A-M and O-P). Early in the G2-M transition, FM4-64 labeled endocytic vesicles follow the emerging EMTs labeled with GFP-MAP4, as shown in single median section (A) and 3D-projection (B). The marked rectangle in (C) is zoomed in for (D-I). FM4-64 labeled endocytic vesicles preferentially internalize from the cortical areas approached by the GFP-AtEB1 labeled EMT plus ends (D-F) (see additional file 6: Movie 6), and oryzalin-induced microtubule depolymerization disrupts their internalization routes (G-I) (see additional file 7: Movie 7) whereas the internalization paths are recovered after oryzalin removal (J). (K) Close-up of GFP-AtEB1 marked EMT plus ends bridging the NE and PPB. Note that during PPB narrowing, FM4-64 labeled endocytic vesicles preferentially internalize from the cortical areas approached by the GFP-AtEB1 (see additional file 8: Movie 8). Formation of an FM4-64 labeled cortical belt at the PPB site (labeled with GFP-MAP4) is shown in a single median section (L) and in 3-D projection (M). (N) 3-D projection of GFP-Ara7 labeled endosomes exhibiting an endosomal belt at preprophase. (O) Both FM4-64 labeled endosomes and ST-YFP labeled GAs form a cortical belt at the PPB site. (P) GFP-MAP4 labeled EMTs connecting the nucleus to the PPB intersect FM4-64 labeled vacuoles. Time is indicated in minutes. Bars represent 7 μm in A, B, J, L, M, 10 μm in C, N-P and 5 μm in K.

**Figure 5:**
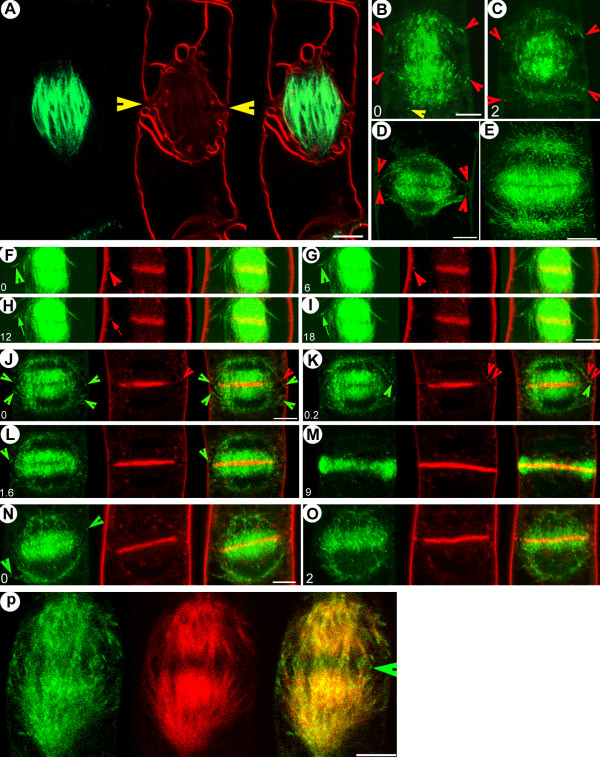
**PM targeted EMT plus ends probe the areas occupied by the preceding PPB and align the cell plates for proper docking at the parental walls**. Green: GFP-MAP4 (in A, F-I), GFP-AtEB1 (in B-E, J-O and P). Red: FM4-64 (in A, F-O), YFP-MAP4 (in P). (A) Discontinuity of the vacuolar structures in the preceding PPB site (arrowheads) is maintained at the spindle stage, as visualized with FM4-64 labeled vacuoles and GFP-MAP4 labeled microtubules.(B-C) At the onset of the phragmoplast stage, GFP-AtEB1 labeled EMT plus ends (red arrowheads) originating from the former spindle poles grow towards the cortex (see additional file 9: Movie 9). Occasionally, they grow towards the polar areas (yellow arrowhead). (D) GFP-AtEB1 labeled EMT plus ends (arrowheads) are attracted to the cortical areas marked by the preceding PPB. At late telophase, the distance through which GFP-AtEB1 labeled EMT plus ends reach towards the cortex is reduced. (E) 3-D projection showing GFP-AtEB1 labeled EMT plus end trajectories directed towards the cortex, which are different from the main phragmoplast structure. GFP-MAP4 labeled EMTs (F-I) or GFP-AtEB1 labeled EMT plus ends (J-M) continue to reach the cortex at the former PPB site and display close proximity to FM4-64 labeled endosomes (red arrow and arrowheads). These endosomes display movement towards the minus end of these EMTs. (N-O) GFP-AtEB1 labeled plus end growth of EMTs (arrowheads) towards opposite sides of the cortex is maintained during cell plate and phragmoplast tilting (see additional file 10: Movie 10). (P) Enrichment of GFP-AtEB1 labeled microtubule plus ends (arrowhead) but not of YFP-MAP4 labeled microtubular parts at the phragmoplast midline. Time in F-I is given in seconds while that in J-O is indicated in minutes. Bars in A-O represent 8 μm while that in P represents 10 μm.

The implications of the observation, which differentiated between intracellular motility and intracellular compartmentalization, became apparent when we investigated the localization pattern for FM4-64 labeled endocytic vesicles in relation to the microtubules. During interphase, FM4-64 labeled endocytic vesicles were randomly localized in the cell (data not shown), but early in the G2-M transition they started coaligning with emerging EMTs (Figure 4A, B). In GFP-AtEB1 transformed cells, these endocytic vesicles displayed internalization paths along the EMT trajectories and their appearance coincided with the cortical sites approached by the EMT plus ends (Figure 4C–F; see also additional file 6: Movie 6). Oryzalin-induced microtubule depolymerization immediately affected endocytic vesicular internalization, with complete disruption of their internalization routes (Figure 4G–I; see also additional file 7: Movie 7). When the oryzalin was washed out, the reformed EMTs again approached the cortex and the internalization of the endocytic vesicle traffic resumed (Figure 4J). Most importantly, during PPB maturation the endocytic material aggregated at the cortical areas occupied by the PPB and approached by the radiating EMT plus ends (Figure 4K; see also additional file 8: Movie 8). Consequently, the endocytic vesicles formed a cortical belt loosely co-localizing with the microtubular PPB (Figure 4L, M). Support for this observation comes from a recent electron microscope study analyzing the membrane architecture of the PPB, which revealed an accumulation of clathrin-coated and non-coated pits specifically in the PPB regions [32]. Moreover, the activity of an endosomal marker protein Ara7 (Rab5 homologue from *Arabidopsis*), is known to be up-regulated during mitosis [33], and a similar PPB belt was observed using GFP-Ara7 labeled endosomes (Figure 4N). Furthermore, the endosomal band we observed co-localized with a band comprising Golgi bodies (Figure 4O) [34]. It is noteworthy that during PPB maturation, the EMTs, which connect the nucleus to the cortical PPB, prohibit a continuous vacuolar structure and thereby create a cytoplasmic area proximal to the PPB (Figure 4P). This cytoplasmic area occupied by the EMTs at PPB maturation is still maintained at the spindle stage (Figure 5A). It has been proposed that the actin-depleted zone (ADZ), which appears during PPB breakdown and is also maintained throughout cytokinesis, participates in regulating the division plane, since actin disruption before ADZ formation affects the cell division planes [35]. We speculate that the lack of actin prohibits further transport of continuously endocytosed material, contributing to the formation of a coherent endosomal belt, for it has been shown that plant endosomal trafficking is mostly actin-based [36].

### EMTs radiating from the former spindle poles attain a geometrical conformation correlating with cell-plate navigation and tilt-correction

Following observations on microtubule and organelle behavior during the early stages of mitosis, we analyzed events at later stages. Immediately after chromosomal separation at anaphase, the EMTs emanating from the region occupied by spindle poles 'probed' the cell cortex (Figure 5B, C; see also additional file 9: Movie 9) while exhibiting unidirectional growth at speeds of 8.52 ± 1.23 μm/min (n = 45). During late telophase, these EMTs mainly probed the cortical areas previously occupied by the PPB (Figure 5D). In addition, the EMTs originating from the non-facing surfaces of the daughter nuclei appeared fewer than the phragmoplast microtubules and occasionally exhibited growth trajectories towards the cell poles (Figure 5E). Because of the remarkable co-incidence between the EMTs and endocytosed material during preprophase, we investigated whether these EMTs approaching the cortex co-localized with FM4-64-labeled endocytic material during telophase. Such co-localization was observed both for GFP-MAP4 (Figure 5F–I) and GFP-AtEB1 (Figure 5J–M) labeled EMTs which were approaching the former PPB sites. During cell plate expansion, the EMTs appeared to navigate the cell plate and to align it to establish a clear line of division (Figure 5N, O; see also additional file 10: Movie 10). EMT plus ends within the phragmoplast midline (where the cell plate subsequently formed) were labeled strongly with GFP-AtEB1 but less strongly with YFP-MAP4, indicating the polarity of the phragmoplast microtubules, oriented with their plus ends towards the developing cell plate (Figure 5P).

In budding yeast, cytokinesis is delayed until the spindle is properly positioned, but in a mutant for Bim1 (EB1 homologue) this delay is abolished, resulting in abnormal cell division [37]. This suggests that Bim1 has an important role in sensing and positioning the spindle. Cytokinesis in plants often begins with tilted spindles, indicating the absence of a spindle alignment checkpoint in plant cells. However, this spindle tilting is corrected later during the progression of cytokinesis, which may indicate the involvement of a positioning sensor and correction mechanism in plant cells. We propose that the EMTs initiating from the spindle poles and approaching the cortex (Figure 5B–O) are involved in this sensor and tilting mechanism. Though different scenarios can be evoked for the correction mechanism, in each case a specialized cortical reference site would be required. We therefore investigated the effect of an auxin efflux inhibitor, NPA, which has been shown to block both vesicular trafficking and internalization of plasma membrane-localized proteins [36] without directly affecting microtubules or actin filaments [38].

### Polarity inhibitor induces abnormal PPBs and shifts cell division planes

**Figure 6:**
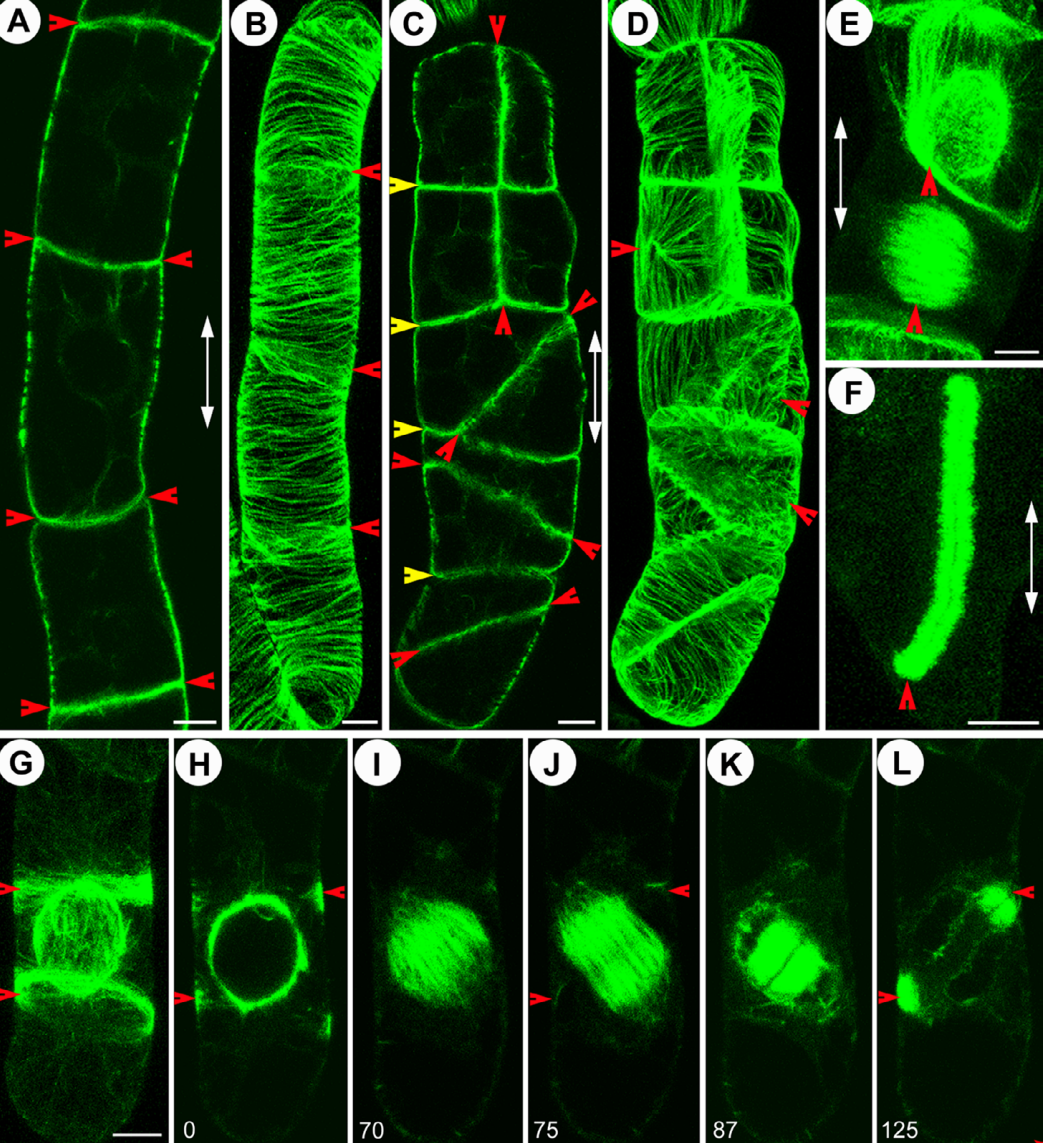
**NPA induces abnormal PPBs and altered cell divisions**. (A-B) Normal anticlinal cell divisions (arrowheads in A) and transverse organization of cortical microtubules in NPA untreated cells. (C-D) Inclined and periclinal cell divisions (red arrowheads in C) with altered organization of GFP-MAP4 labeled cortical microtubules (red arrowheads in D) in NPA treated cells. A, C show single median sections and B, D show 3-D projections. Note that the first round of cell division (yellow arrowheads) is normal and a shift in the cell division planes occurs in the second round. (E-F) Formation of periclinal PPBs and spindles (arrowheads in E) and periclinal phragmoplasts (arrowhead in F). Bidirectional arrows in A, C, E and F show the long axes of the cells. (G-L) NPA treatment sometimes causes formation of two separate PPBs (arrowheads in G) equidistant from the nucleus, which results in tilted spindle formation (I) and phragmoplast initiation (J), phragmoplast growth (K) and cell plate docking (L) at sites marked by either of the PPBs (arrowheads) (see additional file 11: Movie 11). G shows 3-D projection and H-L show single median sections. Bars represent 10 μm and time is indicated in minutes.

Prolonged NPA treatment in BY-2 cells caused inclined and periclinal cell divisions (Figure 6C) instead of normal anticlinal cell divisions (Figure 6A), as in tobacco VBI-0 cells [39]. Cells with aberrant division planes also exhibited major alterations in interphase cortical microtubule alignments in the daughter cells (Compare Figure 6A, B with Figure 6C, D). NPA treatment caused formation of abnormal PPBs (Figure 6E) that resulted in inclined spindles (Figure 6E) and phragmoplasts (Figure 6F), resulting in shifted division planes. In some NPA-treated cells, two separate PPBs were observed and the inclined cell plate was attached to the parental cell walls, with either end docking at one of the places marked by these two PPBs (Figure 6G–L; see also additional file 11: Movie 11). Closer observation of this two-PPB situation revealed a preferential attachment of the cell plate at the PPB sites connecting with the largest number of EMTs (Figure 6H). These results, together with observations from other laboratories, implicate a link between the intracellular establishment of polarity, endocytosis, the placement of initial PPBs and the final cell division planes.

## Discussion

In mammalian and yeast cells, a microtubule plus end-mediated 'search and capture' mechanism has been credited with positioning and aligning the spindles [17,19] and determining the plane of cell division [40]. Our observations suggest that EMTs in plant cells may behave similarly to establish and regulate the cell division plane. Interestingly, it has been shown previously that injuries caused by inserting microneedles at these specific cortical sites, probed by EMTs during cytokinesis, affect cell plate alignment [41].

In mammalian and yeast cells, EB1 binds to adenomatous polyposis coli (APC). In epithelial cells, APC is mainly found at specialized cortical sites [42], providing a planar cue [43]. In mammalian cells, microtubule-mediated APC delivery to specialized cortical sites has been demonstrated [44,45]. A mechanism for attaining polarity cues is proposed for mammalian cells, according to which the EB1-labeled microtubule plus ends target to the specialized APC-marked cortical sites [46]. A similar role in attracting EB1-labeled microtubular plus ends towards the cortex has been attributed to Kar9p in budding yeast [17] and to Moe1 in fission yeast [47]. Moreover, it has been suggested that LIS1 is a regulated adapter between CLIP170 and cytoplasmic dynein at sites involved in cargo-microtubule loading and/or the control of microtubule dynamics [48].

Interestingly, plants seem to possess homologues for APC, Kar9p and Moe1, and the plant cytoskeletal-related Tonneau2 [49] protein contains a LisH domain present in LIS1 (our unpublished results based on the NCBI search engine). In maize, the Tangled1 (which is distantly related to the APC) mutant displays altered PPBs, spindles and phragmoplasts and shifted cell division planes. Furthermore, Tangled1 expression and its microtubule localization correlate with the cell division stage [50]. In mammalian cells, EB1 is required for microtubule tip-specific localization of APC but not vice versa. In the absence of EB1, APC localizes all along the microtubule lengths [50,51]. In addition, APC assembly in the cortical clusters is EB1-independent but depends on the existence of the armadillo domain [52]. In plants, it remains to be determined how EB1 localizes in the Tangled1 mutant and vice versa. From parallels in the mammalian cell literature one might hypothesize that Tangled1-mediated EB1 targeting to specialized cortical sites regulates the cell division planes in plants. However, there are several problems with this hypothesis. For instance, Tangled1 contains only the microtubule binding domain and lacks both the EB1 binding and the armadillo domains of APC [52]. Inversely, AtEB1 possesses an APC interaction domain [23] and a unique C-terminal acidic tail [22], and armadillo domain-containing proteins exist in *Arabidopsis *[53]. This leaves open the possibility that EB1 interacts with other PM-localized basic protein(s). In addition, plants lacking Tonneau2 fail to assemble PPB [4] and Tonneau2 possesses a LisH domain. From the parallel mammalian cell literature one might hypothesize that the LisH domain in Tonneau2 may mediate EMT plus end cortical interactions. Hence it will be interesting to determine how EB1 localizes in Tangled1 and Tonneau2 mutants and vice versa.

In mammalian cells, many proteins have been shown to associate in a microtubule plus end complex, which has been described as a plus end raft [54]. As with lipid rafts, protein concentration at the distal ends may allow a cascade of interactions in the restricted area of a microtubule plus end. This may, in turn, control the dynamic behavior of this cytoskeletal network and its anchoring to other structures [54]. An alternative to this would be that EMTs, by interpreting cell geometry and polarity cues, deposit protein(s) at the PPB, which subsequently attracts the phragmoplast microtubules. Conversely, the connection between EMT plus ends and endocytosis may indicate a role for localized endocytosis in modifying the PM architecture, which may transmit the memory for re-attracting EMT plus ends during cytokinesis. We consider that the polarity inhibitor NPA affects PPB formation by modulating endocytosis, as it does not affect microtubules but interferes with endocytosis and with polarity-based EMT plus end targeting to specialized areas of the PM. Thus, feedback loops, comprising polarity establishment-endocytosis-microtubule plus end guidance-further endocytosis, appear to be essential for defining and creating planes of cell division in plant cells.

## Conclusion

In conclusion, our results suggest that in mitotic plant cells, EMT plus ends may act as cell shape/polarity sensing and orienting machines by their sustained cortical targeting, as shown for yeast [15,55]. EMTs in premitotic plant cells are bundled and bidirectional, as reported very recently in fission yeast by analysis of the EB1 homologue mal3p [56], indicating evolutionary conservation of the processes involved in defining cell division planes. Importantly, we show that at preprophase the targeting of EMT plus ends coincides with endocytosis events to establish a plant-specific cortical endocytic belt. During cytokinesis, this same belt again interacts with the EMT plus ends of the expanding phragmoplast to ensure proper cell plate navigation and docking. Our results reveal a link between the position of EMT plus ends, the establishment of intracellular polarity and the localization of endocytosis that is essential for the regulation of cell division planes in plants.

## Methods

### Plant material and growth conditions

Tobacco BY-2 cells were cultured and transformed as reported previously [9].

### Construction of reporter genes

The construction of GFP-MAP4, YFP-MAP4, YFP-CLIP170 and GFP-AtEB1 was described previously [9,22]. GFP-Ara7 in vector pBSIIKS+ [33] was excised with HindIII-XbaI and sub-cloned into the binary vector pBINPLUS. STtmd-YFP in vector pMON [57] was digested with PstI -SmaI and cloned into the binary vector pCAMBIA 1390.

### Fluorescent dyes and drugs

FM4-64 (Molecular Probes) dissolved in water was applied to the BY-2 cells at 2 μM final concentration for 5 min. The cells were washed with BY-2 medium to remove excess dye and were observed immediately. Alexa 633 (Molecular probes) and Mitotracker (Molecular probes) dissolved in water were also applied at 2 μM final concentration and the cells were observed immediately. Stock solutions of taxol (Sigma-Aldrich), latrunculin B (Sigma-Aldrich) and NPA (Sigma-Aldrich) in DMSO were applied to the cells to give final concentrations of 10 μM, 10 μM and 50 μM respectively. Stock solutions of oryzalin (Greyhound Chromatography and Allied Chemicals, Merseyside, UK) were prepared in ethanol and used at 10 μM final concentration.

### Live cell analysis

For live cell analysis, the Zeiss CLSM510 system implemented on an inverted (Axiovert 100) microscope was used. The microscopy system, sample preparation, single wavelength scanning, image processing and movie generation were as previously described [9]. Dual color imaging was performed using dual excitation/emission scanning in multitracking mode. For GFP /YFP dual scanning, we used excitation/emission combinations of 458 nm/ BP 475–525 for GFP and 514 nm/ BP 530–600 for YFP, in combination with the HFT 458/514 primary and NFT515 secondary dichroic splitters. For GFP/ FM4-64 dual scanning, we used excitation/emission combinations of 488 nm/ BP 505–550 for GFP and 543 nm/ LP585 for FM4-64, in combination with the HFT 488/543 primary and NFT545 secondary dichroic splitters. For GFP/ Alexa dual scanning, we used excitation/emission combinations of 488 nm/ BP 505–550 for GFP and 633 nm/ LP650 for Alexa, in combination with the HFT UV/488/543/633 primary and NFT545 secondary dichroic splitters. All filters were from Zeiss. For time-lapse analysis, images were obtained at 1–10 s time intervals. All experiments were repeated 3–5 times. Acquired images were processed using LSM510 Image Browser version 3.0 (Zeiss Corp.). Maximum projections were obtained from 0.5 μm spaced serial optical sections and were exported as TIFF files. For time-series scans, all the images were exported as time-series TIFF files. The exported images were processed with Adobe Photoshop version 5.0 (Adobe Systems Inc.). For individual plant microtubule growth measurements, all time scans were analyzed in the animation mode of LSM510 Image Browser 3.2 (Zeiss Corp.) by marking the single ends of individual microtubules in each image by a zoom function and tracking them for several minutes. The shortest displacement of the plus ends resolvable in this analysis was 0.1 μm. The time values were obtained from the respective frame times in the time-lapse. Thereafter, the data were manually transferred into Excel and processed. The microtubule growth histories (kymographs) were obtained by processing the raw data in LSM510 Image Browser 3.2 (Zeiss Corp.).

## Authors' contributions

PD designed the experiments, acquired the data, analyzed and interpreted them and drafted the manuscript. TWJG supervised the research. PD, JM, MH and TWJG participated in manuscript designing, coordination and editing. All authors read and approved the final manuscript.

## Supplementary Material

Additional File 1 GFP-AtEB1 labeled cortical microtubule plus-end dynamics exhibiting bidirectional growth during interphase. The movie is accelerated 80 times. The real time is 4 min. Click here for file

Additional File 2 GFP-AtEB1 (green) labels growing plus ends of YFP-MAP4 (red) decorated microtubules exhibiting microtubule guidance and bundling. The movie is accelerated 90 times. The real time is 3.75 min. Click here for file

Additional File 3 GFP-AtEB1 (green) colocalizes with YFP-CLIP170 (red) on growing microtubule plus ends. The movie is accelerated 60 times. The real time is 6 min. Click here for file

Additional File 4 GFP-AtEB1 labeled endoplasmic microtubule plus ends display bidirectional (incoming and outgoing) growth polarity and bundling. The movie is accelerated 110 times. The real time is 6.5 min. Click here for file

Additional File 5 During PPB maturation, GFP-AtEB1 labeled endoplasmic microtubule plus ends radiate symmetrically, mainly in an outward direction from the NE. The movie is accelerated 100 times. The real time is 12 min. Click here for file

Additional File 6 FM4-64 labeled endocytic vesicles (red) preferentially internalize from the cortical areas approached by the GFP-AtEB1 labeled EMT plus ends (green). The movie is accelerated 80 times. The real time is 20 min. Click here for file

Additional File 7 Oryzalin-induced microtubule depolymerization disrupts internalization routes of FM4-64 labeled endosomes. The movie is accelerated at 80 times. The real time is 7 min. Click here for file

Additional File 8 GFP-AtEB1 marked EMT plus ends bridging NE and PPB. Note that during PPB narrowing, FM4-64 labeled endocytic vesicles preferentially internalize from the cortical areas approached by the GFP-AtEB1. The movie is accelerated at 120 times. The real is 18 min. Click here for file

Additional File 9 At the onset of the phragmoplast stage, GFP-AtEB1 labeled EMT plus ends originating from the former spindle poles grow towards the cortex. The movie is accelerated 60 times. The real time is 3 min. Click here for file

Additional File 10 GFP-AtEB1 labeled plus end growth of EMTs towards opposite sides of the cortex is maintained during cell plate and phragmoplast tilting. The movie is accelerated 60 times. The real time is 2 min. Click here for file

Additional File 11 NPA treatment sometimes causes formation of two separate PPBs equidistant from the nucleus, resulting in tilted spindle formation and phragmoplast initiation, growth and cell plate docking at sites marked by either of the PPBs. The movie is accelerated 550 times. The real time is 120 min. Click here for file
